# Seroprevalence of meningococcal serogroup C bactericidal antibodies in the Portuguese population, a decade after vaccine introduction in the National Immunisation Programme

**DOI:** 10.1371/journal.pone.0250103

**Published:** 2021-04-15

**Authors:** Paulo Gonçalves, Emma Sáez-López, Sofia Carneiro, Maria João Simões

**Affiliations:** 1 National Institute of Health Doutor Ricardo Jorge, Lisboa, Portugal; 2 European Programme for Public Health Microbiology Training (EUPHEM), European Centre for Disease Prevention and Control, Stockholm, Sweden; University of Minnesota College of Veterinary Medicine, UNITED STATES

## Abstract

**Background:**

The incidence of invasive meningococcal disease due to serogroup C (MenC) decreased in Portugal since the introduction of the conjugate vaccine (MCC) in the free market in 2001 and in the National Immunisation Plan in 2006. Considering the potential waning of the antibody response reported in the literature, the different vaccination schemes that were used in our country over the past decade, and that *Neisseria meningitidis* serogroup C continues to circulate, the Portuguese population may currently be at increased risk of infection. In the absence of national data, we evaluated the seroprotection level of the Portuguese population against MenC, in order to identify the protected fraction of the population and ponder on the necessity of a booster dose of the MCC vaccine.

**Methods:**

We measured serum bactericidal antibody levels against MenC in a representative sample of the population (n = 1500) aged 2–64 years who participated in the 2015/2016 National Serological Survey.

**Results:**

A total of 31.1% (466/1500, 95%CI: 29–33%) of the individuals studied were protected against MenC. The geometric mean titre was 6.5. The proportion of seroprotected was particularly low in children aged 2–4 years (<16%) who received a single dose of the vaccine at 12 months of age (vaccination strategy since 2012). The proportion of seroprotected was higher (44.7% to 53.5%) in adolescent and young adults (15–24 years of age), resulting from vaccination during the catch-up campaign at 5–15 years of age. The highest protection rates were observed when the vaccine was administered during adolescence.

**Conclusion:**

The small fraction of population seroprotected, combined with the already known waning effect of the antibody response over time, may indicate that the Portuguese population will become progressively more exposed to the risk of infection. Taking in consideration our results, we recommend to change the current vaccination strategy and introduce a booster dose of the MCC vaccine during adolescence.

## Introduction

Invasive meningococcal disease (IMD) is caused by *Neisseria meningitidis*, which is a commensal bacteria in the human nasopharynx. Up to 5%-10% of people may be asymptomatic carriers, with the highest rates observed in adolescents and young adults [1, 2]. Occasionally, the bacteria crosses the mucosal barrier into the bloodstream, from where it may penetrate the haemato-encephalic barrier, pass through the cerebrospinal fluid and invade the subarachnoid space causing meningococcal meningitis [3–6]. The bacteria may also multiply in the blood-stream and originate septicaemia or septic shock associated with disseminated intravascular coagulation and cardiovascular failure [7]. There are 12 serogroups of *N*. *meningitidis*, which are based on its capsular polysaccharides. However, most of the invasive infections are due to only six of them: A, B, C, W, X and Y [8, 9].

IMD is associated with high, 8%-15%, case fatality ratio, and 10%-20% of survivors will suffer from long-term sequelae [1]. Its incidence and epidemiology are influenced by bacterial virulence factors and by the host susceptibility, which is related with the level of serum bactericidal antibodies (SBA), IgG and IgM, and with antibodies present in the nasopharyngeal mucosa [10, 11]. The incidence varies geographically, ranging from <0.5 to 0.9 cases per 100 000 population in North America and Europe, to 10 to 1 000 cases per 100 000 population in the African meningitis belt [1, 12]. In 2018, the recorded incidence rate in Europe was 0.63 cases per 100 000 population [13]. Age-specific incidence rates were highest in infants (8.34 cases per 100 000 population) and young (1–4 years-old) children (2.38 cases per 100 000 population), and a second smaller peak occurred in adolescents and young adults (15–24 years-old, 0.94 cases per 100 000 population) [13]. In Portugal, the incidence rate for IMD in 2018 was 0.62 cases per 100 000 population, similar to the European average [13]. However, the age-specific rates were higher than the European average, reaching 20.87 and 3.54 cases per 100 000 population in infants and young children, respectively [13]. Advances in health management and control policies have contributed to the decrease in the overall incidence over the last decade, and IMD is now considered rare in EU/EEA countries [14]. Nevertheless, IMD remains a major public health issue due to the high severity of the disease, relatively high case-fatality ratio and long-term sequelae [14, 15].

Immunisation is achieved naturally by colonization, disease, or by vaccination. Indeed, the development of meningococcal serogroup C conjugate (MCC) vaccines is the greatest advance in the control and prevention of the disease due to *N*. *meningitidis* serogroup C (MenC), since the proportion of individuals with serum bactericidal antibody (SBA) activity to meningococci, induced by the vaccine, is inversely related to the incidence of disease [11]. In the early 90s, many countries experienced outbreaks of MenC, mainly due to the clonal complex ST-11, which is particularly virulent and easily transmitted [16]. The MCC vaccine was introduced in several European countries from 1999 onwards, which dramatically altered the epidemiological pattern of the disease [17, 18]. This vaccine induces the production of SBA and avoids nasopharynx colonization, thus inducing herd protection [19–21]. In Portugal, MCC vaccine was introduced in the free market in November 2001 and administered by paediatricians, according to the recommendations of the Portuguese Society for Paediatric Infections following the increasing tendency in the number of cases that had been reported since 1998 [22]. The vaccine was administered between 2002 and 2005 as a series of 2–3 doses within the first year of life followed by a booster dose during the second year. At the end of 2005, estimated vaccine coverages for the cohorts between 1997 and 2004 (children between 1 and 8 years-old) ranged from 39% to 69%, respectively [22]. In January 2006, the MCC vaccine was included in the Portuguese National Immunization Programme (NIP) and recommended as a 2+1 dose series at 3, 5 and 15 months of age, for children born after September 2005 [23]. In order to reach the objective of having all birth cohorts since 1989 vaccinated, the NIP 2006 included two retrospective vaccination campaigns. One was targeted to children born between October 2004 and September 2005 (4 to 15 months old), who in 2006 were considered eligible for vaccination according to 3-dose scheme. The second was a single-dose catch-up campaign conducted during 2006 and 2007 targeting children born between January 1989 and September 2004, aged 2 to 18 years, not yet vaccinated or who had received one dose before 12 months-old. By 2012, vaccination coverage estimates had improved to between 80%, for the 1989 cohort, and over 95%, for the 2006–2010 cohorts [24]. In January 2012, the 2+1 dose scheme was replaced by a single dose at 12 months of age, with no booster dose afterwards on the basis of two assumptions: i) vaccination of adolescents in the 2006–2007 catch-up campaign reduced the asymptomatic carriers to a residual number, ensuring the protection of yet unvaccinated children and, ii) the protection endures in time [25]. The first assumption was based on the experience reported in other industrialised countries [26–28], whereas the second had no scientific basis. Vaccination coverage estimates for the 2012 to 2015 cohorts remained at 98% (General-Directorate of Health, personal communication).

Data from the national surveillance system for IMD [22, 29] showed a steady reduction in the global incidence of IMD in Portugal from an average of 3.38 cases per 100 000 inhabitants in 2000–2003 to 0.41/100 000 in 2016, demonstrating the success of the MenC vaccination campaigns in reducing the incidence of the disease in the country. However, many countries have observed falling levels of immunity against MenC over time, showing the need of a booster dose during adolescence in order to maintain herd protection and, in doing so, protect the vulnerable population, particularly non-vaccinated children (younger than 12 months) [21, 30, 31]. In addition, there is evidence of a rapid decrease of immunity after vaccination in infants, even after the administration of 3 doses within the first year of life, resulting in a high proportion of children (1–4 years and even 5–13 years of age) being non seroprotected against MenC disease [21, 31, 32]. These individuals will not be contributing to the indirect protection of the unvaccinated population.

Data on *N*. *meningitidis* serogroup C antibody persistence are important in indicating the level of protection of the population, and the point at which booster doses become necessary [21]. However, studies on the prevalence of SBA against MenC have not been performed in Portugal. The aim of this study was to identify the protected fraction of the Portuguese population by quantifying MenC SBA levels in individuals aged 2–64 years, ten years after the introduction of the vaccine in the NIP. This will allow a reflection on the implications of the different vaccination schedules adopted since the introduction of the vaccine in the country and consider the need of a booster dose of the vaccine, in order to ensure the protection of the whole population through herd protection.

## Materials and methods

### Study population

A national wide cross-sectional and retrospective study was performed, with a convenience sample of the Portuguese population 2–65 years of age (n = 1500) who participated in the 2^nd^ National Serological Survey, conducted in Portugal during 2015 and 2016 for the vaccine preventable diseases included in the National Immunisation Programme and coordinated by the Portuguese National Institute of Health (INSA) in Lisboa, Portugal [33]. For that National Serological Survey, a total of 4866 individuals were recruited taking in consideration the representativeness of the population and the prevalence of the individual diseases. One serum specimen was collected for each recruited individual, which was anonymised and sent to the National Reference Laboratory (NRL) for Vaccine Preventable Diseases at INSA and stored at −80 °C. In the context of the National Serological Survey, anonymised demographic and social data were collected. However, only the date of birth was included in this study. Information on individual immunization status (including MCC vaccination) was not available. An Excel format database was obtained from the 2^nd^ National Serological Survey curators, from which serum specimens were randomly selected for this study, using the RAND() function of the software.

### Sample size

In the absence of national data on MenC seroprevalence, stratified sampling was performed according to the estimated prevalence of SBA to MenC from a similar study, conducted in England by Ishola and colleagues [31] a decade after introduction of the MCC vaccine in the country, for seven birth cohorts. These were divided according to the vaccination schedules adopted in Portugal: 2012–2014, 2006–2011, 2002–2005, 1997–2001, 1988–1996, 1982–1987 and 1952–1981. The expected proportion (%) of individuals with protective (≥8) SBA titres for each of these seven cohorts was obtained from that shown by Ishola and colleagues for individuals within the corresponding age category at the time of sampling (S1 Table). Sample size was calculated for each birth cohort strata using the web-based open-source programme OpenEpi (Dean AG, Sullivan KM, Soe MM. OpenEpi: Open Source Epidemiologic Statistics for Public Health. www.OpenEpi.com, update 2013/04/06), with a 95% confidence interval (95%CI) and a margin of error of 6%. A total of 1500 sera were included in this study.

### Serological assays

Serological assays were performed at the NRL for *N*. *meningitidis* at INSA. Sera was tested using a standardised SBA assay as described by Maslanka *et al*. [34] using 3–4 week rabbit complement (Pel-Freez Biologicals, Rogers, AR, USA), the *N*. *meningitidis* serogroup C strain C11 phenotype C:16:P1.7–1,1, and the Anti-Meningococcal Serogroup A/C Reference Serum Pool (CDC 1992, NIBSC code 99/706, National Institute for Biological Standards and Controls, WHO International Laboratory for Biological Standards, Hertfordshire, England). Titres were expressed as the reciprocal of the final serum dilution giving ≥50% bacterial mortality at 60 min. The lower and upper limits of detection were titres of 4 and 2048, respectively. For geometric mean titre (GMT) analysis, titres <4 and ≥2048 were assigned a value of 2 and 2048, respectively. Titres ≥8 are considered protective against MenC disease [35].

### Statistical methods

Analysis was performed on Stata12.1 (StataCorp. 2011. Stata Statistical Software: Release 12. College Station, TX: StataCorp LP) using a significance level of 5% for all statistical tests. The proportion of seroprotected individuals (titre ≥8) and corresponding exact binomial 95%CI were calculated. SBA GMTs and corresponding 95%CI were also calculated.

### Ethical approval

This study was approved by the Ethical Commission of the National Institute of Health Doutor Ricardo Jorge.

## Results and discussion

Overall, 31.1% (466/1500, 95%CI: 29–33%) of the Portuguese population aged between 2 and 64 years in 2015/2016 had a protective (≥8) SBA titre. The GMT was 6.5 (95%CI: 6–7), which is below the protective titre.

**Fig 1:**
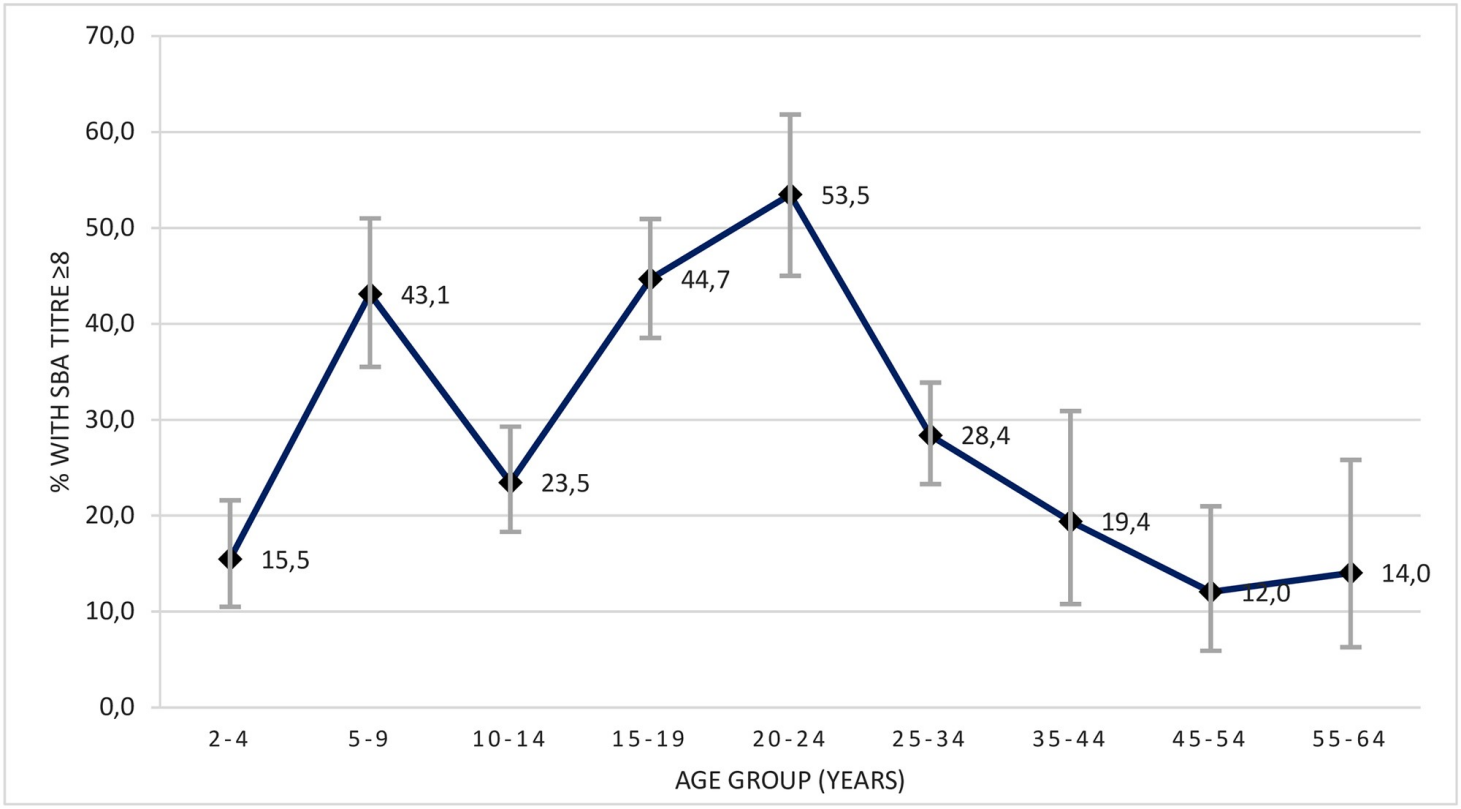
Percentages of serum samples with protective (≥8) SBA titres and 95%CI, by age group, in Portugal, 2015/2016.

The analysis of the proportion of seroprotected individuals by age group is shown on Fig 1. The group of young children between the ages of 2 and 4 years, who received a single dose of the MCC vaccine at 12 months of age, had the lowest percentage of individuals with protective antibody titres, 15.5% (95%CI: 10–22%). The GMT in this age group was 3.4 (95%IC: 3–4), one of the lowest observed in our study.

In contrast, older children 5–9 years of age, included in the 2006 routine vaccination scheme at 3, 5 and 15 months, registered one of the highest proportions of seroprotected individuals, 43.1% (95%CI: 36–51%), with the GMT increasing to 10.8 (95%CI: 8–15).

The second lowest proportion of seroprotected individuals within the vaccinated population was observed in the age group of 10–14 years, with 23.5% (95%CI: 18–29%) of individuals showing protective antibody titres and a GMT of 4.4 (95%CI: 4–5). These young adolescents were either vaccinated with 2 or 3 doses within the first year of life and a had booster dose in the second year (depending on the age of first administration), or had been eligible for a single-dose in the catch-up vaccination campaign of 2006–2007 when they were aged from 12 months to 4 years.

The percentages of seroprotected individuals, as well as GMT, were highest in older adolescents 15–19 years (SBA≥8: 44.7%, 95%CI: 39–51%; GMT: 12.4, 95%CI: 9–16) and young adults 20–24 years (SBA≥8: 53.5%, 95%CI: 45–62%; GMT: 19.1, 95%CI: 13–28). These individuals had been eligible for single-dose catch-up vaccination during the 2006–2007 campaign, when they were 5 to 15 years-old.

Within the unvaccinated population (over 35 years), 15.0% (95%CI: 10–21%) were seroprotected against MenC disease, with a GMT of 3.4 (95%CI: 3–4).

Table 1 shows the proportion of seroprotected individuals and GMT, by vaccine schedule. In spite of the high (98%) vaccination coverage achieved in the 2012–2014 birth cohort, who was vaccinated with a single dose of the MCC vaccine at 12 months of age, only 14.1% (95%CI: 9–21%) of the individuals were found to be seroprotected 1 to 3 years later in 2015/2016. The GMT for this cohort in that period was below the threshold for seroprotection (3.4, 95%CI: 3–4). In a similar study conducted in the England, Ishola and colleagues [31] also found that the lowest proportion (33.9%; 95%CI: 27–42) of seroprotected individuals was observed in children who were eligible for a single dose of the vaccine in their second year of life in a catch-up campaign, 1–4 years after vaccination. That proportion decreased even further, to 15.6% (95%CI: 5–33%), a decade after vaccination.

**Table 1:** Proportion of seroprotected (titre ≥8) individuals and geometric mean titre, by vaccine schedule, in Portugal, 2015/2016.

Vaccine schedule	Birth cohort	Estimated vaccine coverage	Sample (n)	Seroprotected (Titre≥8)		GMT (95%CI)
				(n)	% (95%CI)	
1 dose at 12 months of age (NIP 2012)	2012–2014	98%^a^	156	22	14.1 (9–21)	3.4 (3–4)
2+1 doses at 3, 5 and 15 months (NIP 2006)	2006–2011	95%^b^	201	82	40.8 (34–48)	9.7 (7–13)
1–3 doses within the first year of life with a booster during the second year^c^	2002–2005	96%^b^	195	44	22.6 (17–29)	4.3 (3–5)
1–2 doses within the first year of life and 1 dose in the second year^d^						
1 dose at 2–4 years of age^e^						
1 dose at 5–18 years of age^e^	1997–2001	94%^b^	263	108	41.1 (35–47)	10.1 (8–13)
	1988–1996	88%^b^	263	138	52.5 (46–59)	17.5 (13–23)
1 dose at <19 years^f^	1982–1987	Unk	211	40	19.0 (14–25)	3.4 (3–4)
Not vaccinated	1952–1981	NA	211	32	15.2 (11–21)	3.4 (3–4)
Total			1500	466	31.1 (29–33)	6.5 (6–7)

^a)^ Source: General-Directorate of Health, Portugal, personal communication;

^b)^ average for the period based on reference [24];

^c)^ Pre-NIP vaccination, depending on the vaccine brand and date of first administration, by medical initiative;

^d)^ 2006 campaign for children born October 2004 to September 2005 (4–15 months);

^e)^ 2006 catch-up campaign for children born between 1989 and September 2004 (2–18 years);

^f)^ Pre-NIP, by medical initiative.

NIP: National Immunisation Programme; GMT: geometric mean titre; CI: confidence interval; Unk: unknown; NA: not applicable.

MCC vaccination induces the production of both plasma cells and memory B cells by a T-cell dependant response [36]. Without further boosting and considering the waning effect in the level of antibodies produced in primary vaccination [32], it becomes clear that the one-dose scheme at such a younger age is ineffective in inducing enduring, or even short-term, protecting SBA levels to MenC disease. If the Portuguese population follows the same pattern observed in the England, it will be expected that the 2012–2014 birth cohort may, within a decade after vaccination, have only a residual level of seroprotection. Therefore, protection in this cohort will be dependent on the indirect protection achieved by reducing the carrier state or the incidence of the disease resulting from herd protection [36].

In contrast with the one-dose scheme at 12 months, 40.8% (95%CI: 34–48%) of children who were vaccinated with a 2+1 dose scheme at 3 and 5 months with a booster at 15 months of age (born between 2006 and 2011) were still protected 4 to 10 years later, in 2015/2016. However, GMTs in this group were only slightly above the threshold for seroprotection (9.7; 95%CI: 7–13). A recent study from Findlow and colleagues [32] reported that children, who were vaccinated with a 2+1 dose scheme at a similar age of 3, 4 and 12 months, showed a lower proportion (31.6%, 95%CI: 24–40%) of seroprotection 1–3 years after vaccination, and an even lower proportion after additional 5 years (19.1%; 95%CI: 11–30%).

It is recognised that booster doses of the conjugated vaccine stimulate memory B cells to further differentiate into plasma cells and more memory B cells, thus contributing to immunologic memory [36]. However, data suggests that these 2+1 dose schemes at an early age may not be sufficient in producing enduring protection to MenC disease, particularly if we also take into consideration the waning effect of the antibody response within the first decade after vaccination [31, 32]. As such, the Portuguese 2006–2011 birth cohort, who in 2015/2016 may still have played a protective role for the younger population will, in a short period, expectedly fall below the level of seroprotection and no longer contribute to the protection of younger age groups.

Our study shows that the highest proportions of seroprotected individuals, 41.1% (95%CI: 35–47%) and 52.5% (95%CI: 46–59%), were observed in older adolescents and young adults born between 1997–2001 and 1988–1996, respectively, who were eligible for the one-dose catch-up vaccination campaign in 2006/2007 when they were older children (5–10 years) and adolescents (11–19 years of age), respectively. GMTs in these cohorts ranged from 10.1 (95%CI: 8–13) to 17.5 (95%CI: 13–23). Even though the vaccination coverages may have ranged from 80–96% in 1989–1996 to 92–97% in 1997–2001 [24], this campaign appears to have been quite effective in inducing a protective immunity level almost a decade after vaccination. Similar observations were made in England [31, 32] where catch-up campaigns aimed at primary and secondary school-aged children resulted in 56.1% (95%CI: 47–65%) and 55.8% (95%CI: 48–63%) of individuals still protected one decade later while adolescents and young adults, respectively, with GMTs ranging from 27.5 (95%CI: 18–43) to 28.3 (95%CI: 19–42). Even though the waning effect of immunity over time was also observed in these studies, over 44% of these individuals remained seroprotected 15 years later, with GMTs higher than 15 and, in consequence, still contributing to the indirect protection of younger age groups. Such data clearly demonstrate the advantages of vaccinating later in childhood or during adolescence.

Individuals in the 2002–2005 birth cohort were vaccinated with different schemes and with different vaccination coverages (ranging from 94% in 2005 to 97% in 2004), for a mean vaccination coverage of 96% for that period. According to data from the Portuguese Health Authorities, 61–70% of children born between January 2002 and September 2004 were vaccinated with 2–3 doses within the first year of life with a booster during the second year, following the 2+1 or 3+1 schemes that were recommended by the vaccine manufacturers at that time [22]. An additional 28–32% were vaccinated with a single dose of the vaccine in the 2006/2007 catch-up campaign, when they were aged 2–4 years [23]. For children born between October 2004 and September 2005, they were either vaccinated with at least 2 doses of the vaccine within the first year of life and a booster during the second year (recommended scheme by vaccine manufacturers), or with a single dose in the second year of life (in the context of the 2006 catch-up campaign for <2 years-old children). Although we could not find data in the literature that could quantify the proportion of children vaccinated by each of those vaccination schemes, considering the vaccination coverages for the Jan2002/Sep2004 group we assume that the majority would have been vaccinated with at least 2+1 doses, and a smaller proportion vaccinated with one dose in the catch-up campaign. These different schemes may justify the fact that the proportion of seroprotected individuals for the 2002–2005 cohort in 2015/2016, 22.6% (95%CI: 17–29%), was higher than that observed for the single-dose scheme at ≥12 months of age given to the 2012–2014 birth cohort (14.1%, 95%CI: 9–21%), but a lower than the one observed with the 2+1 dose scheme in children born in 2006–2011 or with the one-dose catch-up later in life (40.8%, 95%CI: 34–48%).

Individuals born between 1982 and 1987 were not targeted for any vaccination campaign, but an unknown number of them may have been vaccinated with one dose of the vaccine by medical initiative at their adolescence/young adulthood (15–20 years). This may have contributed to a discrete 19.0% (95%CI: 14–25%) level of seroprotection in that group. Also not targeted for any vaccination programme were individuals who were born before 1981 (35+ years at the time of sampling). Although individuals in this group were most probably not vaccinated, there is still some degree of protection within the group (15.2%; 95%CI: 11–21%), that may be attributable to natural infection or colonisation with *N*. *meningitidis* serogroup C during their life.

## Conclusion

We have shown that the proportion of children with protective SBA titres against serogroup C meningococci was low, despite the estimated high vaccination coverage achieved for the MCC vaccine, in Portugal, 2015/2016. Also, only around half of teenagers and young adults, who could confer protection to the younger, were protected. Our study, as well as studies in other countries, demonstrate that vaccination programmes followed by catch-up campaigns targeting teenagers and young adults may contribute to reduce the overall proportion of individuals at risk for MenC disease in the population, particularly in children.

Without further boosting, and considering the waning effect of the antibody response observed within the first decade after vaccination, it is expectable that, in time, the Portuguese population may become progressively more exposed to the risk of infection, since *N*. *meningitidis* serogroup C strains continue to be identified from both patients with IMD and carriers (nasopharyngeal and genital).

The use of vaccines is an essential measure in public health for the control of MenC disease. However, vaccination policies must take into consideration not only the target population and its immune status, but also the vaccination schemes. The findings from this study demonstrate the fragility of the current strategy for prevention of MenC disease in Portugal. Therefore, it is recommended that the current vaccination scheme should be revised, aiming at the introduction of a booster dose of the MCC vaccine (or of the tetravalent ACWY conjugate vaccine, considering the fact that the frequency of IMD due to other serogroups of *N meningitidis*, particularly serogroup W, has been increasing in Portugal since 2017) during adolescence, and that the efficacy of the prevention strategies continue to be monitored in periodical seroprevalence studies.

## Supporting information

S1 Table Estimated sample size for each birth cohort strata. Serum samples were taken from the 2015–2016 National Serological Survey.
(DOCX)
