# Supplementary material for: Seroprevalence of meningococcal serogroup C bactericidal antibodies in the Portuguese population, a decade after vaccine introduction in the National Immunisation Programme
Source: PLoS One. 2021 Apr 15;16(4):e0250103. doi: 10.1371/journal.pone.0250103 (PMC8049472; doi:10.1371/journal.pone.0250103)
Supplement: S1 Table — Serum samples were taken from the 2015–2016 National Serological Survey. (DOCX) [file pone.0250103.s001.docx]

| S1 Table. Estimated sample size for each birth cohort strata. Serum samples were taken from the 2015-2016 National Serological Survey. | | | | | | |
| --- | --- | --- | --- | --- | --- | --- |
| Birth cohort | Corresponding birth cohort in the study by Ishola and colleagues [31] | | Proportion (%) with protective (≥ 8) SBA titre in the study by Ishola and colleagues [31] | | Estimated sample size |  |
| 2012-2014 | 2006-2008 | | 31% | | 229^a^ |  |
| 2006-2011 | 1999-2005 | | 25% | | 201 |  |
| 2002-2005 | 1995-1998 | | 24% | | 195 |  |
| 1997-2001 | 1990-1994 | | 56% | | 263 |  |
| 1988-1996 | 1982-1989 | | 56% | | 263 |  |
| 1982-1987 | Pre-1982 | | 27% | | 211 |  |
| 1952-1981 |  |  | 27% | | 211 |  |
|  | |  | | Total | 1573^b^ |  |
| ^a^ Estimated sample size for 2012-2014 was 229, but due to lack of sera only 156 we included; ^b^ Final sample size was 1500 sera. | | | | | | |
